# The NOVA system can be used to address harmful foods and harmful food systems

**DOI:** 10.1371/journal.pmed.1004492

**Published:** 2024-11-19

**Authors:** Jean Adams

**Affiliations:** MRC Epidemiology Unit, University of Cambridge, Cambridge, United Kingdom

Food systems include all of the interconnecting processes involved in producing, distributing, and retailing food. Food systems currently contribute around one-third of global greenhouse gas emissions [1], and the unhealthy food and diets they deliver account for around 15% of deaths globally [2]. Both the human and planetary harms of food systems are unequally distributed with less affluent individuals, communities, and countries enduring the greatest impacts [3].

Addressing these problems of harmful foods delivered by harmful food systems requires interdisciplinary research and cross-sectoral policy action. Researchers must focus on both identifying the foods causing most harm to human and planetary health, as well as the aspects of food systems that mean those foods continue to be produced. This demands attention from both life and social scientists.

The NOVA system offers a new way to approach the simultaneous problems of harmful foods and harmful food systems. NOVA categorises foods into 4 groups based on “the *extent* and *purpose* of the industrial processing they undergo” [4] (emphasis added; Table 1). The ultraprocessed foods (UPF) that NOVA identifies are often very convenient and palatable and are branded to create social value that extends beyond their nutritional content and taste. For example, many brands aim to associate themselves with aspirational lifestyles.

**Table 1:** The NOVA classification of foods based on the extent and purpose of the industrial processing they undergo (adapted from [4]).

Group	Name	Extent of processing	Purpose of processing	Examples
1	Unprocessed and minimally processed foods	Industrial processes such as removal of inedible or unwanted parts, drying, crushing, grinding, fractioning, roasting, boiling, pasteurization, refrigeration, freezing, placing in containers, vacuum packaging, or nonalcoholic fermentation; no other food substances added to the original food.	To extend food life, enable longer storage, make preparation easier.	Milled grains, raw meat, fruit and vegetables canned in water.
2	Processed ingredients	Substances obtained directly from group 1 foods or from nature by industrial processes such as pressing, centrifuging, refining, extracting, or mining.	For use is in the preparation, seasoning, and cooking of group 1 foods.	Oils and fats, sugar and salt.
3	Processed foods	Industrial products made by adding salt, sugar, or other substance found in group 2 to group 1 foods, using preservation methods such as canning and bottling and nonalcoholic fermentation.	To increase the durability of group 1 foods and make them more enjoyable by modifying or enhancing their sensory qualities.	Cheese, fruits, and vegetables canned in syrup or brine.
4	Ultraprocessed foods	Fractioning of whole foods into substances that include sugars, oils, and fats, proteins, starches, and fibre. Some of these substances are then submitted to hydrolysis, or hydrogenation, or other chemical modifications. Subsequent processes involve the assembly of unmodified and modified food substances with little if any whole food using industrial techniques such as extrusion, moulding, and prefrying. Colours, flavours, emulsifiers, and other additives are frequently added. Processes end with sophisticated packaging.	To create highly profitable products (low-cost ingredients, long shelf-life, branded products) which are liable to displace all other NOVA food groups.	Carbonated soft drinks, margarines, “instant” noodles.

UPF have become common concepts in both the life and social sciences. Life scientists have documented associations between consumption of UPF and morbidity and mortality from numerous causes and have explored biological mechanisms explaining these associations. Social scientists have documented the tactics used by the small number of transnational companies that dominate the global UPF supply to drive demand and minimise attempts at regulation such as front-of-pack labelling and restrictions on advertising.

By including both “extent” and “purpose” in its definition, the NOVA system inherently incorporates this duality of life and social science paradigms. By providing a unifying concept around which we can address the key questions of both which foods, and which aspects of food systems, cause the most harm, this duality is perhaps the key added value that the NOVA system has to offer. Unfortunately, this may also lead to a paradigm clash between life science and social science uses of the concept, focused on the “extent” of industrial processing and the “purpose” of industrial processing, respectively. This potential for paradigm clash may prevent the real value of the NOVA system from being achieved.

Much of the current academic, policy, and media attention on UPF focuses on the extensive epidemiological literature reporting links between the extent of UPF consumption and health harms. There are primary studies, systematic reviews, and umbrella reviews reporting these harms [5]. In the substantial commentary that accompanies these studies, there is debate about the specificity and reliability of the categorisations made using the NOVA system, whether we can interpret evidence from prospective cohort studies as causal, the limitations of the one major randomised controlled trial published to date [6], and the biological mechanisms that might link UPF consumption with health harms [7]. Much of this debate mirrors wider discussions in epidemiology about exposure misclassification and causality and may simply reflect a new concept reinvigorating old arguments. However, it is also possible that those with most to gain from continued scientific uncertainty about the health harms of UPF (i.e., the companies that make and sell them) deliberately emphasise this uncertainty in media commentary.

Alongside this life science research activity, there is also a growing literature on the purpose of ultraprocessing. This social science documents a broader range of mechanisms through which UPF might maintain harmful food systems. These include how UPF are marketed to encourage overconsumption, the extensive profits that large multinationals generate from UPF sales that drive them to minimise and avoid regulation to maintain the status quo, and the many indirect effects that their operations can have that they are not directly responsible for addressing. These indirect effects range from increasing healthcare costs to address the health harms of UPF overconsumption, to changing norms of acceptable governance through behind-closed-doors lobbying undermining transparent democracy [8]. There are also concerns about the impact of these multinationals on the process and direction of science itself via their potential impact on public and scientific debate, as described above, as well as through direct funding of scientists. This social science literature concludes that there are harms of UPF dominated food systems over and above any direct health harms from UPF consumption. It reflects the wider literature on the “commercial determinants of health” [9] and attracts similar criticism—that the authors are ideologically driven and antiprofit, antigrowth, and anticapitalist.

Rarely do these 2 bodies of literature come together. Those coming from more life science perspectives sometimes critique the “purpose” aspect of NOVA as “unscientific”—it is impossible to objectively infer “purpose” from something like industrial processing. Those from a more social science perspective counter that science is rarely entirely objective, that the narrow focus on seeking to achieve more and more certain estimates of effect sizes on health outcomes ignores the wider, systemic harms of UPF, and that being distracted by investigation of biological mechanisms detracts from action based on known political economic mechanisms.

Stuck in the midst of this scholarly endeavour are real people. Qualitative literature tells us that citizens consume UPF for the reasons we are all familiar with—they can be cheap, convenient, tasty, and quick. They help us to solve everyday food problems, allowing us to focus our attention on other priorities. Whether or not they cause long-term harms, UPF have many short-term benefits. While some decry the “anti-UPF lobby” as food snobs seeking to deprive the masses of affordable nourishment, others point out that the majority of citizens in many countries would welcome regulation to make it easier to eat less processed alternatives [10].

What then should we do about UPF? Certainly, we should continue to explore the 2 separate strands of life and social science research. Understanding biological mechanisms could be instructive for making sense of accruing evidence that only some categories of UPF are associated with health harms [11] and may identify routes to creating healthier UPF. We also need to better understand the environmental impacts of UPF [12]. But we should not ignore the wider harms of UPF-focused food systems. We need clearer understanding of the influence of the UPF industry on the direction of science and policy and how this can be countered. Furthermore, reformulated UPF are still UPF and even if they have fewer direct health harms, they may still generate all the indirect harms of an overconcentrated, profit-first food system that resists regulation of price, advertising, and labelling. We need much better understanding of how to disrupt food systems to support healthier, more sustainable eating.

We also need more and better work exploring the interactions between these life and social scientific viewpoints of UPF. Such interdisciplinarity requires open, honest, and sometimes difficult dialogue to overcome differences in language, methods, and ways of knowing. But it may well help make progress on the trade-offs that these dual views on UPF might require policymakers to grapple with. For example, what should the policy response be to (a hypothetical) conclusion that some UPF are more harmful to human health, but less harmful to planetary health, compared to less processed alternatives? Or that UPF create substantial costs to society in terms of healthcare, but benefits to individuals in terms of food prices? The answers to these questions require us, as societies, to be clear what our values are and how we want to act on them.

By drawing on both life and social scientific paradigms, the NOVA system and the concept of UPF offer a framework that helps us remember that while individual foods may cause harm, so too can wider food systems. NOVA is unlikely to offer the final word on what can be done to disrupt current food systems to support people and planet, but it may help draw in a wider, multi-, and interdisciplinary group of actors who can work together to create positive change.
